# Supplementary material for: The cost of oral cancer: A systematic review
Source: PLoS One. 2022 Apr 21;17(4):e0266346. doi: 10.1371/journal.pone.0266346 (PMC9022815; doi:10.1371/journal.pone.0266346)
Supplement: Attachment — Submitted filename: response to reviewer_07022022.pdf [file pone.0266346.s002.pdf]

Dear Editor,

We thank you for the opportunity to have our manuscript **PONE-D-21-31795**, entitled "The cost of oral cancer: a systematic review", evaluated for publication by Plos One. After analyzing all the comments made, we are happy to implement the suggestions to improve the manuscript, as described below. The manuscript line numbers cited on each answer below are in accordance with the file "manuscript_with_track_changes".

Yours sincerely,

The Authors

Table 1: Summary

**Questions**	**Reviewer 1**	**Reviewer 2**
Is the manuscript technically sound, and do the data support the conclusions?	Yes	Yes
Has the statistical analysis been performed appropriately and rigorously?	N/A	N/A
Have the authors made all data underlying the findings in their manuscript fully available?	No	Yes
Is the manuscript presented in an intelligible fashion and written in standard English?	Yes	Yes
Review Comments to the Author	See below	See below

Reviewer 1, comment 1: "I thank the authors for their submission. This is a nice systematic review of the economic burden of oral cancer. From a methodologic perspective, this work is rigorous and adheres to many best practices including a completed PRISMA checklist as well as an a priori protocol, available and registered on PROSPERO."

Our response: We would like to thank you for your consideration and time spent reviewing our manuscript.

REVIEWER 1, MAJOR COMMENTS

Reviewer 1, comment 2: "My main reservation is with the concept of cost of illness studies more broadly. Cost of illness studies are frequently criticized for not being grounded in welfare theory. They are descriptive and felt to have limited utility in informing resource allocation, particularly in comparison to other forms health economic evaluation (eg. cost-effectiveness analyses). Nonetheless, by comprehensively outlining direct and indirect cost components across various countries, I do think this review may assist with future cost-effectiveness studies in oral cancer. The authors touch on some of

these issues briefly in the limitations section, though I do think the limitations of COI should be discussed more explicitly."

Our response: Thank you for your comment. In fact, cost-of-illness studies cannot be used to inform resource allocation. For this purpose, a full economic evaluation is required (cost-effectiveness, cost-utility, cost-benefit, or cost-minimization), by which at least two mutually exclusive strategies are compared in terms of their costs and health consequences.

However, we have not claimed the results of our systematic review should be used in resources allocation. As we reported in the introduction, we just sought to identify the economic burden of the disease. On this basis, the use of cost-of-illness is not a limitation of our results. It was what we planned for.

Cost-of-illness is very important to inform the economic burden of a disease, which is a summary of the costs of a particular disease to society, health system or other point of view (perspective of the study). This information can be used to estimate avoidable costs if policies/programmes are implemented to reduce the prevalence of the disease. Moreover, cost-of-illness can also inform the distribution of the economic burden between types of costs (healthcare costs and productivity losses) and between levels of care (prevention, diagnosis, treatment). This information can be used to understand how a society is funding a specific disease. Cost-of-illness can also be used to inform priority setting, by providing estimates of how big a problem is in terms of costs. We included those points in the introduction (page 3, lines 61-62; page 4, lines 65-66), as reported below.

"Although clinical and epidemiological aspects of oral cancers are well-documented in the literature, there is a lack of evidence on the economic burden of oral cancers worldwide. Cost-of-illness studies can provide information on the monetary consequences of a disease or condition, including healthcare costs and productivity losses, and its impact on societal or public health expenditure [6]. This information can be used to estimate avoidable costs if policies/programmes are implemented to reduce the prevalence of the disease. When available, it also can inform costs stratified by stages of the disease. In the United Kingdom, average treatment cost for oral cancer can range from I$ 3,343 in the early stages to I$ 24,890 in the advanced stages [7]. Cost-of-illness can also be used to inform priority setting, by providing estimates of how big a problem is in terms of costs [8]. Moreover, gathering infor-mation on costs may encourage decision makers to implement strategies for detecting and screening populations at high-risk of developing oral cancer, particularly by comparing costs at different stages of the disease. To the best of our knowledge, up to now there are no systematic reviews that synthesize evidence on the economic burden of oral cancer. The objective of this study is to provide a comprehensive systematic assessment of the economic burden of oral cancer based on available evidence worldwide."

NOTE: Due to the inclusion of a new reference (8), all subsequent references have been reordered.

Reviewer 1, comment 3: "The quality of included studies was poor. On pages 31 and 32 the authors very briefly outline what constitutes a high-quality COI study in oral cancer. This should be further elaborated on. I think many of these concepts might not be well

known to the head and neck oncology audience. A concise but explicit discussion of prevalence vs. incidence approaches, top-down vs bottom up, prospective vs. retrospective etc. might be helpful to the reader."

Our response: As suggested by the reviewer, we included the required information in the Results section of the manuscript (page 32, lines 340 - 343).

"Regarding the resource quantification, most of the included studies used a top-down approach (18 studies), generally obtained by allocating portions of a known total expenditure to a specific disease stratified by type of cost. Only 6 studies relied on individual data (bottom-up approach), generally obtained by multiplying the unit costs by quantities."

and in the Discussion (pages 33 and 34, lines 371 - 388)

"The main characteristics that qualify a COI study are expressed in its methodological definition. These include, among other aspects, the epidemiological approach, costing method and data collection. Incidence-based COI studies should include both direct and indirect costs throughout the life course to outcome. Prevalence-based COIs also include direct and indirect costs over a given period from any stage of the disease. For an acute illness, these two approaches would estimate similar costs. However, for a chronic disease, such as oral cancer, longitudinal incidence-based studies would provide more accurate estimates of the costs of this disease overt time. Considering the costing method for identifying and measuring resources, the COI approach can be micro (bottom up) or macro costing (top down). Using the micro-costing method, costing components and items are measured at the most detailed level possible, with estimated costs per individual, and the selection of a representative sample is recommended to allow external validity or generalizability of the results to a broader population. In macro costing, the total aggregate cost is divided by the number of individuals and can be expressed as an average value. Generally, COI studies that use micro-costing are more accurate, but less generalizable. Regarding data collection, retrospective studies represent a challenge because the data are secondary, generally intended for other purposes (epidemiological or surveillance) and may not be sufficient for a COI study. Most of the studies included in this systematic review did not meet all the items of the instrument used for quality assessment."

REVIEWER 1, MINOR COMMENTS

Reviewer 1, comment 4: "Minor Comments: I appreciate the explicit use of 'PICO' in the development of the research question, though do not think that an abbreviation for population (P) , exposure (E), and outcome (O) are needed in the abstract."

Our response: We agree with your suggestion, which contributes to a clean abstract. The abbreviations were removed from the abstract (page 2, lines 24-26).

Reviewer 1, comment 5: "Minor Comments: - Could the authors explain why other health economic evaluation studies (CEA, CUA etc…) were explicitly excluded? Presumably some of these studies would have tabulated the direct and indirect costs of oral cancer and could potentially have been included?"

Our response: Thank you for raising this question and for the opportunity to clarify it. We excluded full economic evaluation (cost-effectiveness, cost-utility, cost-benefit, or cost-minimization) because they estimate cost for specific strategies under comparison i.e., they do not provide cost estimation for the disease as a whole. For example, a cost-effectiveness of drug A compared with drug B for patient with tongue cancer will considered just costs related to drug A and B; there would be other diagnoses, outpatient and inpatient care incurred by patients not related to drug A and B, thus this kind of studies might bias our estimates from cost-of-illness. Based on that, we opted for excluding them.

Reviewer 1, comment 6: "Minor Comments: -For the Larg and Moss checklist, how did the authors define the threshold of >80% points as 'high quality' and 50-79% as 'medium quality'? Is that an accepted approach?"

Our response: Thank you for the important comment, which alerted us to the arbitrariness in establishing the threshold as high-quality; medium and low quality to meet the items of the quality assessment instrument. Thus, we maintained the percentage intervals of compliance with the items of the aforementioned instrument without establishing a quality judgment. Adjustments in the manuscript was done as below:

In the Methods section: (page 9, lines 187-192)

"Percentage intervals were established for meeting the items of the quality assessment instrument applied to the included studies: >80%; between 79% and 50%; and less than 50%."

In the Results section / Quality assessment: (page 17, lines 250-253)

"The global quality score of the studies, considered as the percentage rate of compliance to the items of the quality evaluation instrument, was 47.8% (SD=10.9). The quality score varied from 38% [20,35] to 66% [19] (Table 3). "

Table 3 was also modified. (page 18)

Reviewer 1, comment 7: "Minor Comments: -Do the authors have any statistical measures of agreement during the article screening phase?"

Our response: Thank you for your question. Kappa statistic was calculated but it has not been included in the manuscript. This information was added:

in the Methods section (page 6, lines 131-135):

"Kappa statistic was calculated to assess agreement between reviewers, in pairs, with a significance level of 5% (p<0.05). The scale of Kappa value interpretation was as following: <0 no agreement; 0 — 0.20 slight; 0.21 — 0.40 fair; 0.41 — 0.60 moderate; 0.61 — 0.80 substantial and 0.81–1.0 perfect. "

and in the Results section (page 10, lines 216-218):

"In the eligibility stage, Kappa coefficient was 0.83 (perfect) between the EAS and VM reviewers; 0.78 (substantial) between EAS and NRD and 0.78 (substantial) between VM and NRD."

Reviewer 1, comment 8: "Minor Comments: -In the results section "€35,642 in a mathematical model estimated for 10 years". Could you briefly explain what the 'mathematical model' was?

Our response: Thank you for your comment. The study (van Aghtoven, 2001) used a probabilistic model to estimate costs over a 10-year period, considering the probability of patients experiences some health states after year 2, year 4 and years 5–10.

These information was added in the result section (page 30, lines 306-309), as follow:

"€35,642 in a probabilistic mathematical model estimated for 10 years [31] in the Netherlands, which presented the health state after year 2, after year 4 and after years 5–10, calculated from the date of the primary diagnosis…"

Reviewer 1, comment 9: "Minor Comments: -Table 2 – How did the authors categorize the study as a 'cost-analysis' vs 'cost-of-illness' study? Could the authors clarify either in the body of the manuscript or as a footnote in the table?

Our response: Thank you for your comment. We included this information as required on Table 2 (page 16).

"*We classified the type of study according to the comprehensiveness of the cost estimation. If the cost estimation was restricted to a small sample, the study was classified as cost analysis, generally a group of patients from one hospital; and if the cost estimation included a city, state or country, the study was classified as cost-of-illness.*"

Reviewer 1, comment 10: "Minor Comments: -Additional files 1 and 2 appear to be missing, though I was able to piece together the search strategy and MESH terms from the published protocol. The only additional file included is the completed PRISMA checklist."

Our response: Additional files 1 and 2 were deposited in Figshare repository with an embargo of two months. I have already published them and they can be accessed by its DOI number https://doi.org/10.6084/m9.figshare.16663537.v1. This information was added in the last page of the manuscript (page 43, line 686)

REVIEWER 2, MAJOR COMMENTS

Reviewer 2, comment 1: "Thank you for inviting me to review this article. This systematic review study identified 24 studies (2001-2021) published around the world on the cost of oral cancer. Both direct and indirect costs were considered. Grey literature and non-English papers were also searched. The included studies mostly followed the standard cost-of-illness approach and adopted a provider (hospital) perspective. Direct medical costs were examined by most studies, while direct non-medical costs and indirect costs associated with premature mortality and work absence were seldom assessed. Four studies have estimated burden of illness using cost per patient over per-capita GDP. These studies found the cost burden of lip, oral cavity, and oropharynx cancers to amount to 18.3%, 74.8%, and 126.8% of the per-capita GDP of various developed nations. This is a well-conducted systematic review with meaningful economic outputs to inform resource allocation in cancer care. I have a few major concerns about the review methodology and other minor suggestions that the authors may want to consider in their revisions."

Our response: We would like to thank you for your consideration and time spent reviewing our manuscript.

Reviewer 2, comment 2: "Major comments: 1. It is unclear how the cost items in Table 4 and lines 151-154 were defined. If the authors want to survey all types of costs associated with oral cancer (i.e., in order to understand the economic burden of oral cancer), why use an a priorly defined list of cost items to deliberately limit the scope of cost? Are these cost items derived from an established costing framework for oral cancers from the literature? If not, the authors should include a section that outlines how they determined and refined a costing framework and cost components which by itself should be a contribution of this review. The authors might want to consult this systematic review on the methodology of defining a costing framework: Clarke K, Klarenbach S, Vlaicu S et al. The direct and indirect economic costs incurred by living kidney donors—a systematic review. Doi: 10.1093/ndt/gfl069. In particular, the authors only considered two types of indirect costs (work absenteeism and premature mortality). What about the indirect loss due to disability or the extra expense of senior care/childcare due to the patient being hospitalized and unable to provide such care? All these questions beg for a costing framework that exhaustively identifies and categorizes all potential cost components of oral cancer before the literature search.

Our response:

Thank you for your comment. We elaborated Table 4 based on the main types of costs: i) direct costs, which are breakdown into two categories (medical costs and non-medical costs); and indirect costs. This first stage was based on the literature, particularly methodological guidelines. In the second stage, we included only cost items that emerged from the studies included in the systematic review. On this basis, it was not our intention to exhaust all cost items, just highlighting what we identified in the included studies.

Reviewer 2, comment 3: "Major comments: 2. The authors can enhance this study significantly by formally defining the key elements of an economic analysis before the literature search. For example, in what way does a cost-of-illness study differ from a cost study (Table 2)? Why is Patterson (2020) a cost-of-illness study (Table 2) if it has only

assessed the indirect costs of premature mortality resulted from oral cancers (Table 4)? Another key concept that begs for clarification is perspective (Table 2). I find it unconventional to categorize “perspectives” into hospital, government, payer, and society. It appears the authors are mixing up two concepts: 1) the provider of care or the setting in which the care occurs, such as hospital; 2) the payer who affords the cost of care, such as the government (public payer) or the society. I would make 2 columns in Table 2 to distinguish these entities and add a formal definition for perspective in the methods section. Furthermore, if Milani (2021) used a government (public payer) perspective, it should not have included any direct non-medical costs (unless the government reimburses for these costs). Same with Han (2010) and Amarasinghe (2019).”

Our response:

We added a new text in the Results section (pages 10, lines 233,234), and as a Table 2 footnote (page 16, lines 245-247), by which we defined the criteria used to classify cost analysis and cost-of-illness studies, as below. Patterson (2020) was classified as cost-of-illness because it provided estimation of premature mortality for several countries.

“We classified the type of study according to the comprehensiveness of the cost estimation. If the cost estimation was restricted to a small sample, the study was classified as cost analysis, generally a group of patients from one hospital; and if the cost estimation included a city, state or country, the study was classified as cost-of-illness.”

We decided to reanalyse the classification of the type of studies and their perspectives (pages 15-16 (Table 2); page 10, lines 233,234 and page 11, lines 236-237). One study (Lairson, 2017, Ref 41) has its type classification changed from cost analysis to cost-of-ilness. Regarding the perspective of studies, some of them had their perspective reclassified. The final four options of perspective of studies were: i) societal, which included direct and indirect costs and/or out-of-pocket costs from patient point of view; ii) government (public payer), included direct costs only; iii) private health insurers (private payer), which included direct costs reimbursed by the private health insurers; and iv) hospital, which included direct cost charged by just one hospital, unless the authors explicitly reported the government or health insurer perspectives. These final reclassified options were added in the Methods section (page 7 and 8; lines 151-152; 158-163).

“The perspective of studies was defined as: i) societal, which includes direct and indirect costs and/or out-of-pocket costs from patient point of view; ii) government (public payer), includes direct costs only; iii) health insurance provider (private payer), which includes direct costs reimbursed by the private health insurers; and iv) hospital, which includes direct cost charged by just one hospital, unless the authors explicitly reported the government or health insurer perspectives.”

Regarding Milani´s study (2021), direct non-medical costs refer to food and transportation subsidized by the government.

In Han’s (2010) study, the author´s mention, in the method/data analysis, that “hospitalizations cost goes to nursing, supportive care and lodging”, which led us to classify the study as from the hospital perspective.

In Amarasinghe’s (2019) study, non-medical costs were estimated by societal perspective, instead of Provider (hospital). This was identified and reclassified (Table 2, pages 15-16), as we reanalysed the classification of the studies perspectives (page 11, lines 236-237).

Reviewer 2, comment 4: “Major comments: 3. It is absolutely essential that a common currency & year (such as 2019 USD) is used throughout the manuscript (and Table 5) to make between-study comparisons and summarization of costs meaningful. Could the authors convert all currencies (and explain how it is converted) to a common currency & year and present both the initial and converted currency? One way to do this is to first inflate the initial currency to 2019 constant values using the national annual CPI and then adjusted to 2019 USD (or another currency) using PPP from OECD. A minor point is to please remove the currency symbols and replace them with the ISO codes for currencies.”

Our response: Thank you for raising this very important question. Despite of being absolutely essential that a common currency and period of time of economic evaluation is used to make between-study comparisons and costs meaningful, we have not converted the studies’ results to a common currency (PPP, for example) because these values are not comparable across studies (Table 5), due to several differences in terms of method employed, data availability, health system funding, health services access etc. If they are not comparable, it does not make sense to convert to a common currency. When values were comparable (Table 6), we applied the PPP conversion. It is worth noting that the rationale of Table 6 is to compare values within each country (GDP per capita and the cost of oral cancer), and not across countries. The heterogeneity of studies may have impacted on our findings, which did not allow a meta-analysis. Also, we agree to remove the currency symbols and replace them with the ISO (ISO 4217) codes for currencies throughout the manuscript.

REVIEWER 2, MINOR COMMENTS

Reviewer 2, comment 5: “Minor comments: Methods Lines 98-99: why explicitly excluding cost-effectiveness/cost-utility studies since they also assessed cost? Studies might not examine costs as their primary objective but nonetheless present useful data. Perhaps the authors want to state that they only included studies whose primary objective was to assess the cost of oral cancer.”

Our response: Thank your comment and as it was also explained to reviewer 1 (comment 5). We excluded full economic evaluation (cost-effectiveness, cost-utility, cost-benefit, or cost-minimization) because they estimate cost for specific strategies under comparison i.e., they do not provide cost estimation for the disease as a whole. For example, a cost-effectiveness of drug A compared with drug B for patient with tongue cancer will considered just costs related to drug A and B; there would be other diagnoses, outpatient and inpatient care incurred by patients not related to drug A and B, thus this kind of

studies might bias our estimates from cost-of-illness. Based on that, we opted for excluding them.

Reviewer 2, comment 6: "Minor comments: Results In Table 1, the authors might want to report more information of cohort from each study (age, sex composition etc) beyond the size of cohort. Difference of the study cohort contributes to the high between-study heterogeneity as well.

Our response: Thank you for your comment. Information on gender and age or age group of samples from each study was included in Table 1 (pages 12-14), if available.

Reviewer 2, comment 7: "Minor comments: Page 19 (Table 4): please revise Table 4 to reflect two major categories of costs (direct vs. indirect costs), and under direct costs the authors can further define direct medical costs and direct non-medical costs.

Our response: Table 4 was revised and formatted to reflect the two major categories of costs (direct vs. indirect costs), (page 21).

Reviewer 2, comment 8: "Minor comments: Page 30 (Table 6): since only 4 studies reported the economic burden of oral cancer as a percentage of per-capita GDP, and all studies were based in developed western countries, I would not state these results as a general finding in the abstract. Furthermore, only one of these 4 studies (Kim 2020) has examined all cost components as defined in Table 4. Then, why is the cost-per-patient estimate from the remaining 3 studies reliable if the authors have already identified them to represent an underestimation? Alternatively, the authors might want to use the estimate of Rezapour (2018) to manually compute the burden of disease as a percentage of total cost in per-capita GDP."

Our response:

Thank you for the comments and suggestions. We agree that the decision to estimate the average cost of oral cancer, considering the heterogeneity of the studies, has to be taken with caution. So, we decided to exclude the percentage GDP average (Table 6, page 32), since it does not represent a summary measure of this set of heterogeneous studies. To appropriate report this, changes were made in the following sections of the manuscript:

In the abstract (page 2, lines 31-32):

"In some developed western countries, the costs of LC, OCC, and OPC reached an average of Gross Domestic Product per capita of 18%, 75%, and 127%, respectively. Inpatient costs for OC and LC were 968% and 384% higher than those for outpatients, respectively."

In the Discussion section (page 33, lines 361):

"In some western countries, the economic burden of OCC and OPC is more than 60% of GDP per capita [23,26,32], reaching 215% of US GDP per capita (OPC).[41] "

Indeed, we decided to use only the information on the cost of oral cancer related to per capita GDP already reported by the authors (4 studies), since most of them do not provide the annual costs of oral cancer to make adjustments considering the inflation of each country, to compute the burden of disease as a percentage of total cost in per capita GDP. Despite Rezapour's study being the only one that presented all components of direct and indirect costs, it does not report a percent of total oral cancer cost as per-capita GDP.
